# Inhibition of PCSK9 enhances the anti-hepatocellular carcinoma effects of TCR-T cells and anti-PD-1 immunotherapy

**DOI:** 10.7150/ijbs.93668

**Published:** 2024-07-15

**Authors:** Weikang Xu, Minli Hu, Xinyu Lu, Yueqiong Lao, Na Ma, Yiyue Wang, Jing Li, Xingyuan Chen, Shiming Liu, Jing Liu, Wei Zhu, Hui Yang

**Affiliations:** 1 Department of Gastroenterology, The Second Affiliated Hospital of Guangzhou Medical University, Guangzhou 510220, China.; 2 Department of Infectious Diseases, Nanfang Hospital, Southern Medical University, Guangzhou, China; State Key Laboratory of Organ Failure Research; Key Laboratory of Infectious Diseases Research in South China, Ministry of Education; Guangdong Provincial Key Laboratory of Viral Hepatitis Research; Guangdong Provincial Clinical Research Center for Viral Hepatitis; Guangdong Institute of Hepatology. Nanfang Hospital, Southern Medical University, Guangzhou, 510515, China.; 3 Department of Hematology, The Second Affiliated Hospital of Guangzhou Medical University, Guangzhou 510220, China.; 4 Guangzhou Institute of Cardiovascular Disease, The Second Affiliated Hospital of Guangzhou Medical University, Guangzhou 510220, China.; 5 Department of Pathology, The First People's Hospital of Foshan, Foshan 528000, China.

**Keywords:** hepatocellular carcinoma (HCC), proprotein convertase subtilisin/kexin 9 (PCSK9), TCR-engineered T cell (TCR-T), low-density lipoprotein receptor (LDLR)

## Abstract

T cells play important roles in antitumor immunity. However, given that the hepatocellular carcinoma (HCC) tumor microenvironment confers resistance to T cell-based immunotherapies, novel strategies to boost T cell-mediated antitumor efficacy are urgently needed for the treatment of HCC. Here, we show that high proprotein convertase subtilisin/kexin type9 (PCSK9) expression was negatively associated with HCC patient's overall survival and markers of CD8^+^ T cells. Pharmacological inhibition of PCSK9 enhanced tumor-specific killing and downregulated PD-1 expression of AFP-specific TCR-T. Inhibition of PCSK9 significantly enhances the anti-HCC efficacy of TCR-T cells and anti-PD-1 immunotherapy *in vivo*. Moreover, PCSK9 inhibitor suppressed HCC growth dependent on CD8^+^ T cells. Mechanically, pharmacological inhibition of PCSK9 promoted low-density lipoprotein receptor (LDLR)-mediated activation of mTORC1 signaling in CD8^+^ T cells. LDLR deficiency was shown to impair cellular mTORC1 signaling and the anti-HCC function of CD8 T cells. On the basis of our findings in this study, we propose a potential metabolic intervention strategy that could be used to enhance the antitumor effects of immunotherapy for HCC.

## Introduction

Primary liver cancer is the sixth most common and fourth most lethal malignancy, and remains a major global threat to human health^1^. Advancements in cancer immunotherapies over the past few decades have altered the landscape of cancer treatment^2^. Current immune-checkpoint blockade agents for hepatocellular carcinoma (HCC) treatment mainly consist of anti-PD-1/PD-L1 and anti-CTLA-4 antibodies^3^-^5^. However, objective response rates of approximately 15% have been obtained for HCC patients treated with single agents^3^, ^4^, ^6^, which can be ascribed to the fact that the effectiveness of programmed death 1 (PD-1) blockade in treating tumors is contingent upon the presence of pre-existing tumor-infiltrating T cells, which are not always available^7^. Within the nutritionally depleted tumor microenvironment, which is composed immunosuppressive cells or cytokines, tumor-infiltrating T cells can be rendered dysfunctional^8^, and consequently, it is imperative to devise innovative strategies that can enhance the antitumor efficacy of T cells.

Cholesterol metabolism plays a pivotal role in the activation, proliferation, and effector function of T cells^9^. Proprotein convertase subtilisin/kexin type 9 (PCSK9), the ninth member of the proprotein convertase family, interacts with the low-density lipoprotein receptor (LDLR) to modulate intracellular and plasma cholesterol levels, thereby regulating cholesterol homeostasis^10^, ^11^. Recent studies have demonstrated that targeting PCSK9 can promote anti-tumor effects by reprogramming cholesterol metabolism and immunological functions in a range of different cancers^12^, ^13^. Encouragingly, the inhibition of PCSK9 has also been shown to enhance the anti-tumor efficacy of T cells in melanoma and colorectal cancer^14^-^16^. These findings thus tend to indicate PCSK9 could serves as both a tumor and a cell metabolic checkpoint molecule. However, it remains unclear as to whether targeting PCSK9 would enhance cell-mediated anti-HCC immune responses. In our previous investigation, we demonstrated the specific recognition and cytotoxicity of AFP_158_-specific (Tet158) TCR-T-cells against human hepatocellular carcinoma (HCC) cells, both *in vitro* and *in vivo*^17^.

In this study, we demonstrate that targeting PCSK9 enhances the anti-HCC effects of the PD-1 antibody and AFP-specific TCR-T cells. Based on these findings, we propose a novel approach for enhancing adoptive T cell treatment and immune checkpoint therapy for HCC.

## Materials and Methods

### Cell culture

The HepG2, luci-hepa1-6, PLC/PRF/5, Huh7 and SNU449 cells utilized in this study were procured from the Cell Bank of the Type Culture Collection and tested negative for mycoplasmas. HepG2, luci-hepa1-6, PLC/PRF/5 and Huh7 were cultured in Dulbecco's modified Eagle's medium (Gibco, USA) supplemented with 10% fetal bovine serum (Gibco, USA) and 1% penicillin-streptomycin (Gibco, USA), SNU449 cells were cultured in Roswell Park Memorial Institute (RPMI) (Gibco, USA) supplemented with 10% fetal bovine serum (Gibco, USA) and 1% penicillin-streptomycin (Gibco, USA), at a temperature of 37°C under a 5% CO2 atmosphere. AFP TCR T-cells were cultured in RPMI 1640 medium (Gibco, USA) supplemented with 10% fetal bovine serum, 1% penicillin-streptomycin (Gibco, USA), HEPES buffer (20 mM) (Gibco, USA), and Twenty units of interleukin-2 (IL-2) (Peprotech, USA).

### Antibodies and reagents

For flow cytometric analysis, we used the following antibodies purchased from Biolegend: anti-humanCD45 (clone: 2D1), anti-human CD4 (clone: OKT-4), anti-human CD8 (clone: RPA-T8), anti-human CD45RO (clone: UCHL1), anti-human CD62L (clone: DREG-56), anti-human PD-1 (clone: A17188B), anti-human Lag-3 (clone: 11C3C65), anti-mouse CD3 (clone: 17A2), anti-mouse CD4 (clone: RM4-5), anti-mouse CD8 (clone: 53-6.7), anti-mouse CD44 (clone: IM7), anti-mouse CD62L (clone: MEL-14), anti-mouse IFN-γ (Clone: XMG1.2), anti-mouse TCRvβ8.3 (clone: H57-597), anti-mouse Granzyme B (clone: QA16A02), anti-mouse Perforin (clone: S16009A). A monoclonal antibody targeting mouse LDLR was procured from Sino Biological Inc, and Invivoplus anti-mouse CD8a (Clone: BP0061) and Invivomab anti-mouse PD-1 (Clone: BE0146) antibodies were purchased from BioXCell. For western blot analysis, we used PCSK9, alpha 1 Fetoprotein, p-mTOR (Ser2448), mTOR, P-S6K (Thr389), S6K, P-4EBP1 (Thr37/46), 4EBP1, and-actin proteins purchased from Cell Signaling Technology. Filipin III (HY-N6718), PCSK9 recombinant protein (HY-P73728) and PF-06446846 (HY-120088A) were purchased from MedChemExpress, and D-luciferin sodium and diL-LDL were purchased from Yeasen.

### *In vitro* T-cell isolation and activation

To isolate primary spleen T cells from C57BL/6 mice, we used an EasySep™ mouse T Cell Isolation Kit (Stem Cell Technologies). Anti-mouse CD3 and CD28 monoclonal antibodies (Biogems) were used to stimulate mouse primary spleen T cells or CD8 T cells at a concentration of 1 μg/mL and administered with twenty units of mouse IL-2.

### HCC cell killing by TCR-T cells

For lactate dehydrogenase (LDH) analysis, we used an LDH-Glo™ Cytotoxicity Assay (Clone: J2380) purchased from Promega. The percentage of AFP-specific TCR-T-cells was determined based on flow cytometry. The TCR-T were co-cultured with HepG2 at an effector-to-target cell ratio of 1:1. After 24 h of co-culture, the cytotoxicity of T cells was assessed by quantifying LDH activity in accordance with the manufacturer's instructions.

### Cell Counting Kit-8 (CCK8) assay

The proliferation of HCC cells was using the CCK-8 assay (Takara, China). Briefly, 3000 cells/well were incubated in triplicate in a 96-well plate, and 10 μL CCK-8 was added to each well after 24 h. After a 3h incubation at 37°C, the optical density value was measured at 450 nm absorbance.

### Transwell assays

Transwell assays were conducted using 5.0 μM pores chambers (Corning, USA). Five million HepG2 cells were seeded in the lower chamber and incubated for 24 hours, followed by seeding of 5 million TCR-T cells in the upper chamber and subsequent incubation for another 24 hours.

### Western blot analysis

For western blot analysis, we obtained the total protein of human CD8 TCR-T or mouse CD8T cells using RIPA lysis buffer. The protein concentrations were determined using bicinchoninic acid assay. Equal amounts of protein were then subjected to sodium dodecyl sulfate-polyacrylamide gel electrophoresis, followed by the transfer of the separated proteins onto nitrocellulose filter membranes. After protein transfer, the membranes were incubated in a Tris-buffered saline/Tween 20 blocking buffer containing 5% bovine calf serum at room temperature for 1 hour. Subsequently, primary antibody was added and the membranes were incubated overnight at 4°C followed by horseradish peroxidase (HRP)-conjugated secondary antibody. The protein signals were visualized using a UVITEC Cambridge imaging system.

### Flow cytometric analysis

For surface staining, the T cells were incubated with Flow cytometry antibodies at 4°C for a duration of 30 minutes. In the case of intracellular cytokine staining, the cells were stimulated with CD3 and CD28 antibody in the presence of Golgi-Stop (Biolegend) for 4 hours and intracellularly stained for interferon-gamma (IFNγ), granzyme B and perforin as described^18^. The acquired data was analyzed using a BD FACSCelesta flow cytometer and FCS Express Software.

### Mouse HCC models

Male C57BL/6j mice aged 6-8 weeks were procured from the Laboratory Animal Center of Southern Medical University (Guangzhou, China), and male NSG mice aged 6-8 weeks were obtained from Vitalstar Laboratory Animal Technology (Beijing, China). Male C57BL/6j LDLR^-/-^ transgenic mice mice aged 6-8 weeks were obtained from Cyagen Laboratory Animal Technology (Jiangsu, China). 5×10^6^ HepG2 cells were inoculated subcutaneously into the flanks of NSG mice, tumor volumes were calculated using the formula length × width × height × 0.5. For orthotopic implantation, the left lobes of C57BL/6j mice were injected with 2×10^6^ luci-Hepa1-6 cells and tumor burden was assessed via *in vivo* bioluminescence imaging. Mice were grouped under the principle of randomization. At least 4 mice per group were used in all *in vivo* experiments reported here. At the end of the experiment, the mice were euthanized and the excised tumors were weighed and photographed.

### Adoptive TCR-T cell transfer and tumor-infiltrating TCR-T cells analysis

After 21 days, the indicated numbers of human TCR-T with 5×10^4^ U human recombinant IL-2 were intravenously transferred into the NSG mice bearing HepG2 tumors. For the detection of tumor-infiltrating AFP TCR-T cells, the tumors were dissociated using Tumor Dissociation Kit (Miltenyi Biotec, Germany) following the instructions and analyzed by flow cytometry.

### Depletion of CD8^+^ T cells *in vivo*

For hepa1-6 orthotopic implantation model, two days before tumor inoculation, 200 µg per of CD8 antibody (Bio X Cell, USA) or rat IgG (Bio X Cell, USA) were intraperitoneal injected into indicated group. Then, CD8 antibody or rat IgG were injected for every 4 days. CD8 T cells in mice blood were monitored by flow cytometry.

### *In vivo* PF-06446846 and antibody Treatment

For hepa1-6 orthotopic implantation model, after 7 days, mice with similar bioluminescence intensity measured based on *in vivo* imaging were randomly divided into different groups and received PBS, anti-PD-1 antibody (Bio X Cell, 200µg per, every four days, i.p), PF-06446846 (MCE, 5mg/kg, every two days, i.p) or PF-06446846 plus anti-PD-1 antibody injection. For HepG2 HCC model, after 20 days, mice with similar tumor size were randomly divided into different groups received PBS or PF-06446846 (5mg/kg, every two days, intratumoral injection).

### Statistical analysis

The means of two groups were analyzed using an unpaired Student's t-test. One-way analysis of variance (ANOVA) was used to determine statistical differences among three or more groups. All data were analyzed using GraphPad Prism 9.0. at the P < 0.05. level of significance.

## Results

### The expression of PCSK9 is associated with T cell cytotoxicity and infiltration in HCC

**Figure 1:**
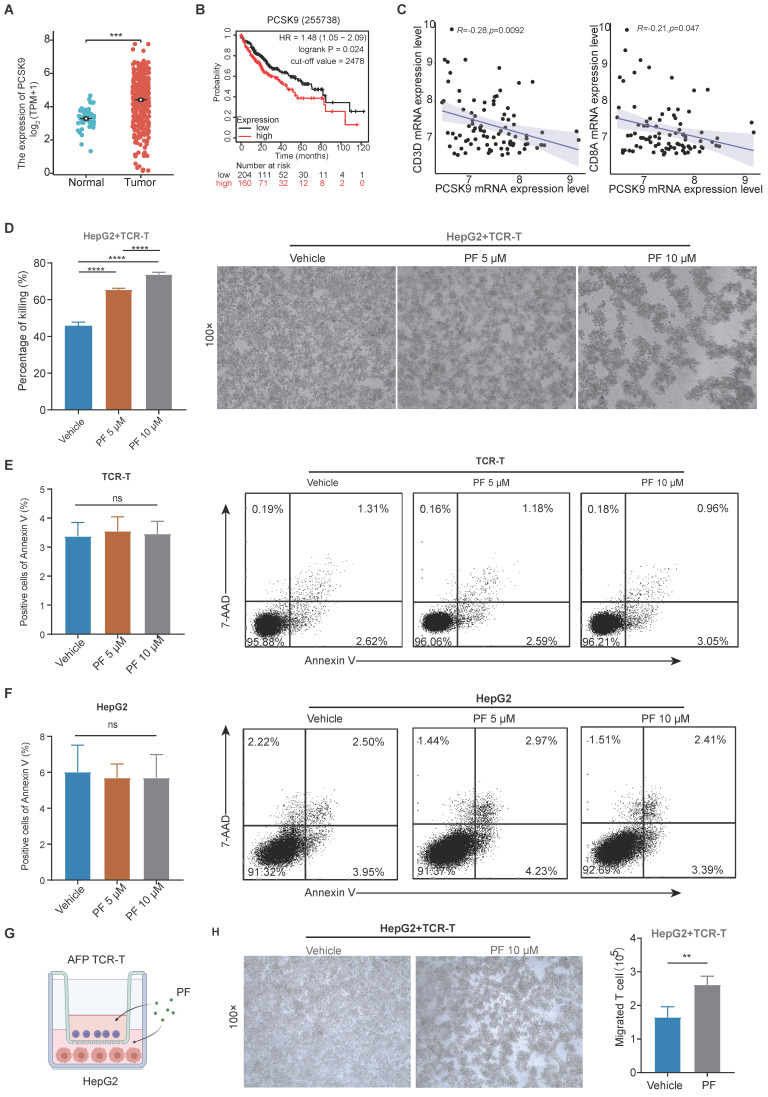
** The expression of PCSK9 is associated with T cell cytotoxicity and infiltration in hepatocellular carcinoma**. (A) The Cancer Genome Atlas analysis of PCSK9 mRNA expression in hepatocellular carcinoma (HCC) tissues and adjacent regions. (B) Kaplan-Meier analysis of overall survival (OS) based on PCSK9 levels of HCC patients. (C) GEO database analysis of the correlation between PCSK9 mRNA expression and T cells in HCC. (D) HepG2 cells were pre-treated with different concentrations of PF-06446846 for 12 h, then co-cultured with TCR-T cells (E: T = 1:1) for 24 h. (E) and (F) Detection of apoptosis using Annexin-V and 7-AAD. (G) Illustration of the co-culture system using HepG2 and AFP TCR-T cells. (H) TCR-T killing of HepG2 cells in the lower chamber of a Transwell system. The number of TCR-T cell showing chemotaxis to the lower chamber. Data are presented as the mean ± SEM. *P < 0.05; **P < 0.01, ***P < 0.001, ****P < 0.0001, NS, not significant.

After analyzing data from The Cancer Genome Atlas (TCGA), we observed a significant upregulation of PCSK9 mRNA expression in HCC specimens compared to adjacent normal liver tissues. (Figure 1A), Kaplan-Meier Plotter analysis revealed that patients characterized by high PCSK9 expression had shorter overall survival (OS) times than those with low PCSK9 expression (Figure 1B). To further investigate the correlation between PCSK9 and T cell in HCC, we analyzed the Gene Expression Omnibus (GEO) HCC database (GSE43619) and found that PCSK9 mRNA expression was negatively correlated with CD3D and CD8A mRNA expression (Figure 1C). PCSK9 mRNA expression was not correlated with CD4, NK and macrophage makers mRNA expression (Figure S1A-C). PF-06446846 is a highly selective inhibitor of translation of PCSK9. Previous studies demonstrated that PF-06446846 can simultaneously target the human and the mouse PCSK9 expression^15^, ^19^, ^20^. PF-06446846 can partially decrease cell viability in HCC cell lines, but not affect TCR-T cells (Figure S2A-B). PF-06446846 can significantly down-regulated PCSK9 expression, but did not affect AFP expression in HepG2 (Figure S2C). Here, we constructed an HLA-A2/AFP_158_ specific TCR-T and verified its function *in vitro* (Figure S2D-G).

Following co-cultured with HLA-A2^+^/AFP^+^ HepG2 cells, we found that PF-06446846 enhanced killing activity of TCR-T cells in a concentration-dependent manner (Figure 1D), but did not affect the apoptosis of TCR-T or HepG2 cells (Figure 1E-F). Furthermore, we evaluated the relationship between PCSK9 and T cell infiltration *in vitro* using a transwell assay system (Figure 1G), which revealed that the PF-06446846 promoted a significant increase in chemotactic behavior of TCR-T cells and enhanced the TCR-T-mediated killing of HepG2 cells in the lower chamber (Figure 1H).

### PCSK9 inhibition increases the effector phenotype frequency of CD8 T cells, although not CD4^+^ T cells

**Figure 2:**
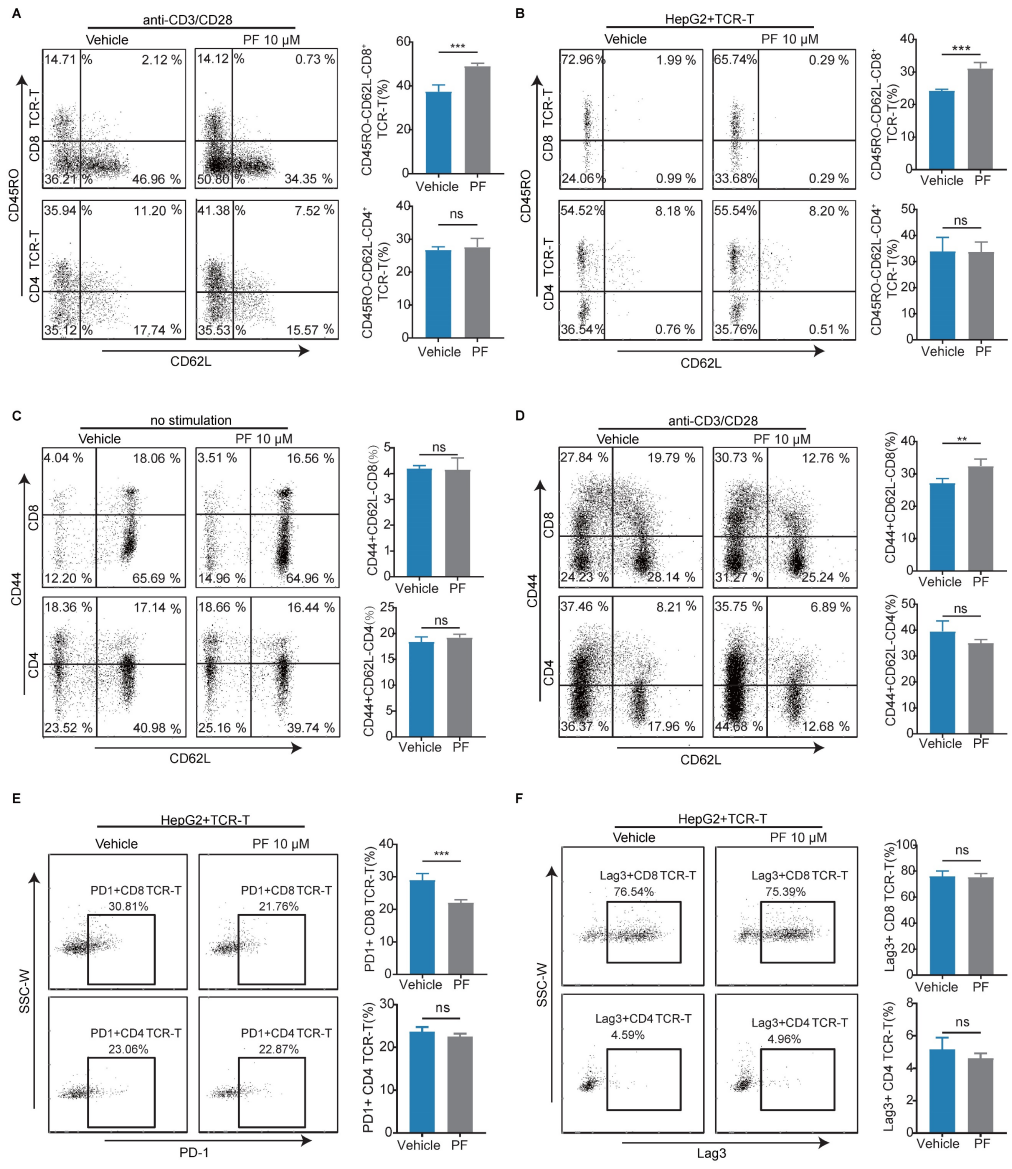
** Anti-HCC effects induced by the inhibition of PCSK9 are dependent on CD8 T cells *in vitro*.** (A) TCR-T cells were treated with or without PF-06446846 under stimulation with 1 μL/mL anti-CD3 and anti-CD28 antibodies for 24 h. Analysis of TCR-T cell memory phenotypes (CD45RO and CD62L) was performed using flow cytometry. (B) TCR-T cells were co-cultured with HepG2 cells for 24 h with or without PF-06446846 (E: T = 1:1). (C) Wild-type (WT) naïve T cells were isolated from mouse spleens, and treated with or without PF-06446846 for 24 h, followed by flow cytometric analysis of memory phenotypes (CD44 and CD62L). (D) WT naïve T cells were treated with or without PF-06446846 under stimulation with 1 μg/mL anti-CD3 and anti-CD28 antibodies for 24 h. (E) (F) TCR-T cells were co-cultured with HepG2 cells for 24 h with or without PF-06446846 (E: T = 1:1), followed by flow cytometric analysis of PD-1 and Lag-3. Data are presented as the means ± SEM. *P < 0.05, **P < 0.01, ***P < 0.001, ****P < 0.0001, NS, not significant.

To further elucidate the impact of PCSK9 inhibition on T cell-mediated anti-HCC activity, we examined memory phenotypes and immune checkpoints. We found that PF-06446846 treatment resulted in a higher percentage of the CD45RO^-^CD62L^-^ effector phenotype than the vehicle, although not the CD4 TCR-T phenotype with anti-CD3/CD28 antibodies (Figure 2A). Having excluded the indirect effects on T cells following the treatment of tumor cells with PF-06446846, we observed similar results (Figure 2B). However, the observed phenomenon did not exert any discernible influence on the phenotype of CD4^+^ T cells. Subsequently, we investigated the memory phenotype of primary T lymphocytes derived from the spleens of C57BL/6 mice, and found that treatment with PF-06446846 promoted an increase the percentage of effector memory CD8 T cells (CD44^+^CD62L^-^) induced by the anti-CD3 and anti-CD28 antibodies (Figure 2C-D). Moreover, the inhibition of PCSK9 was observed to induce a decrease proportion of PD-1^+^CD8 TCR-T cells when co-cultured with HepG2, while exhibiting no significant impact on Lag-3 (Figure 2E-F). Therefore, these findings suggest that the inhibition of PCSK9 facilitates T cell activation during antigen stimulation.

### PCSK9 inhibition promotes CD8^+^ T cell anti-HCC immune responses *in vivo*

**Figure 3:**
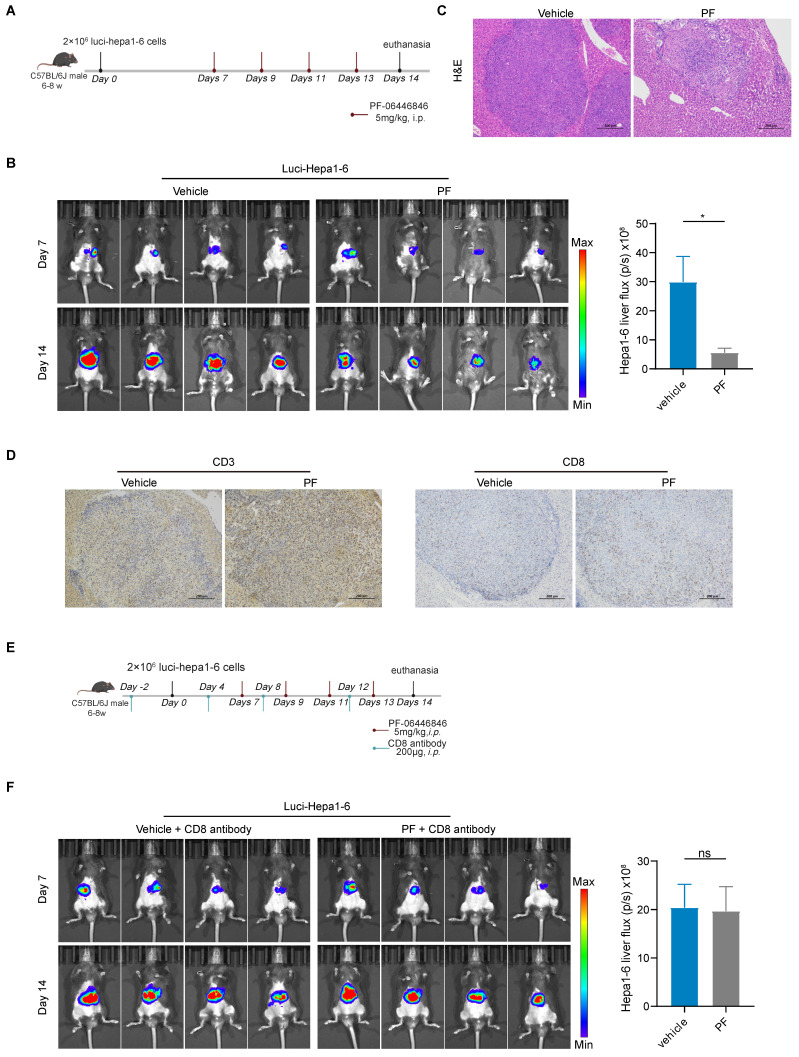
** Anti-HCC effects induced by the PF-06446846 are dependent on CD8 T cells *in vivo***. (A) Treatment with vehicle and PF-06446846 (5 mg/kg) in a luci-hepa1-6 orthotopic implantation mouse model (n = 4 mice per group). The mice were monitored at 7-day intervals and were sacrificed on day 14. (B) Tumor growth was monitored based on bioluminescence values (p/s/cm^2^/sr) and image radiance values were normalized using Living Image (PerkinElmer). (C) Hematoxylin and eosin (H&E) staining. Scale bar = 200μm. (D) CD3 and CD8a immunohistochemical staining. Scale bar = 200μm. (E) Treatment with vehicle and PF-06446846 (5 mg/kg) and an anti-CD8 antibody in a luci-hepa1-6 orthotopic implantation mouse model (n = 4 mice per group). The mice were monitored at 7-day intervals and sacrificed after 14 days. (F) Tumor growth was monitored based on bioluminescence values (p/s/cm^2^/sr) and image radiance values were normalized using Living Image (Perkin-Elmer). Data are presented as the means ± SEM. *P < 0.05, **P < 0.01, ***P < 0.001, ****P < 0.0001, NS, not significant.

To assess the *in vivo* anti-hepatocellular carcinoma efficacy of the PCSK9 inhibitor, luci-Hepa1-6 cells were intraportally injected into the left hepatic lobes of male C57BL/6J mice aged 6 to 8 weeks (Figure 3A), and bioluminescence intensity in the different groups were measured based on *in vivo* imaging. Compared with the vehicle group, PF-06446846 promoted a significant inhibition of HCC tumor growth and tumor weight (Figure 3B-C and Figure S3A), but not affect body weight (Figure S3A). Consistent with the *in vitro* findings, the IHC staining demonstrated an augmented expression of CD3^+^ and CD8^+^ T cells in the PF-06446846 treatment (Figure 3D). To ascertain the CD8 T cell dependence of PF-06446846 therapy *in vivo*, we depleted CD8 T cells using neutralizing antibodies prior to PF-06446846 therapy (Figure 3E). In response to CD8 T cells depletion (Figure S3C), we detected no significant differences between the control and PF-06446846 groups with tumor size, tumor weight and body weight (Figure 3F and Figure S3B).

### Inhibition of PCSK9 promotes anti-HCC immunity by upregulating LDLR in CD8 T cells

**Figure 4:**
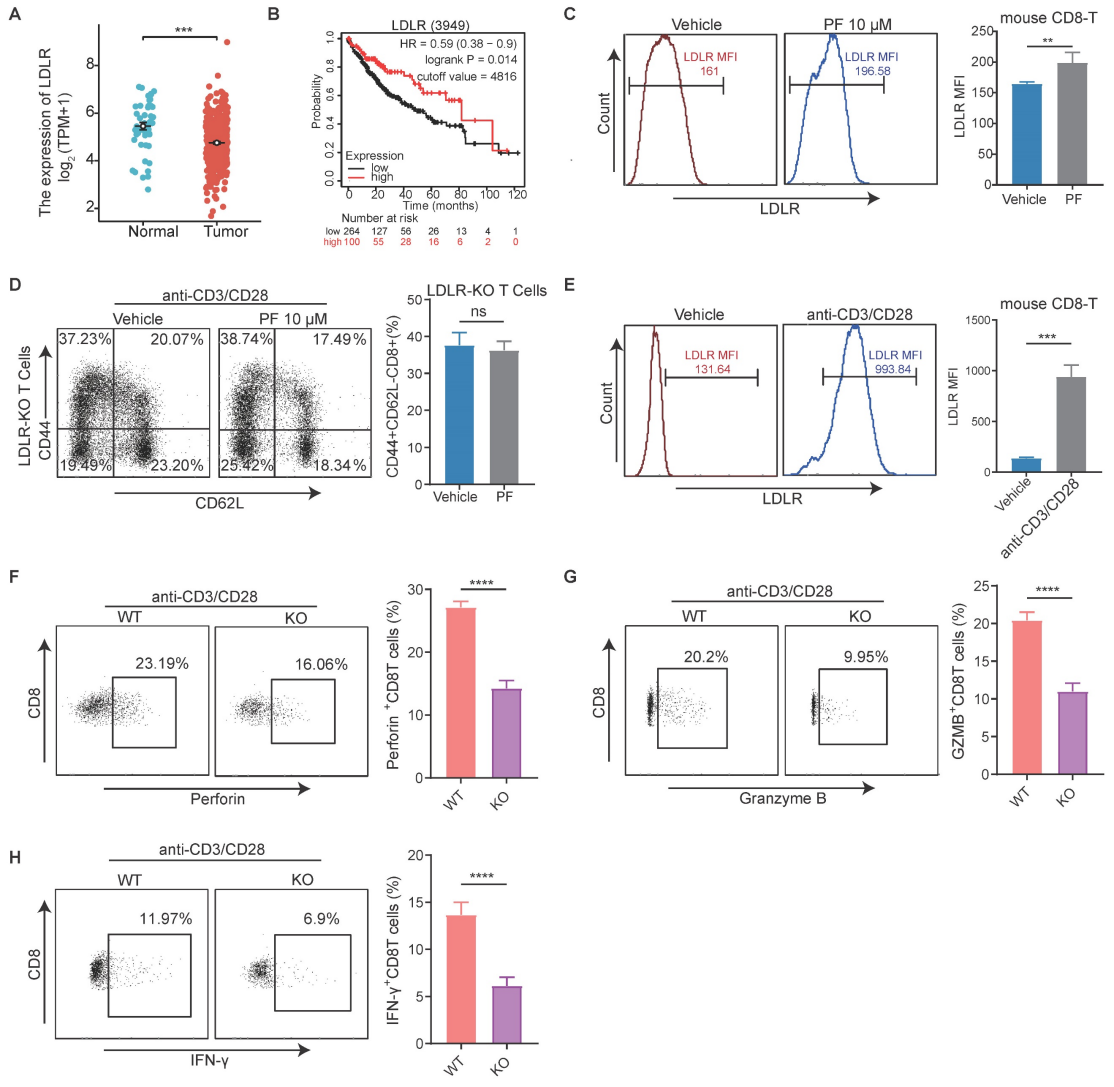
** The inhibition of PCSK9 promotes anti-HCC immunity by upregulating the low-density lipoprotein receptor in CD8 T cells.** (A) The Cancer Genome Atlas analysis of low-density lipoprotein receptor (LDLR) expression in hepatocellular carcinoma (HCC) tissues and adjacent regions. (B) Kaplan-Meier analysis of overall survival (OS) based on LDLR levels in HCC patients. (C) Naïve CD8^+^T cells were treated with PF-06446846. LDLR mean fluorescence intensity (MFI) was analyzed by flow cytometry. (D) LDLR KO Naïve CD8^+^T were treated with or without PF-06446846 under stimulation with 1 μg/mL anti-CD3 and anti-CD28 antibodies for 24 h. and memory phenotypes were analyzed using flow cytometry. (E) Wild-type (WT) CD8^+^ T cells were stimulated with 1μg/mL anti-CD3 and anti-CD28 antibodies for 24 h. LDLR MFI was analyzed by flow cytometry. (F-H) WT CD8^+^ T cells and LDLR KO CD8^+^ T cells were stimulated with 1 μg/mL anti-CD3 and anti-CD28 antibodies for 24 h. Perforin, granzyme B, and IFN-γ production were analyzed based intracellular cytokine staining (ICS). Data are presented as the mean ± SEM. *P < 0.05, **P < 0.01, ***P < 0.001, ****P < 0.0001, NS, not significant.

The primary function of PCSK9 is to enhance the levels of low-density lipoprotein (LDL)-cholesterol (LDLc) by sorting and escorting the LDL receptor (LDLR) to lysosomes^21^. Therefore, we conducted an initial analysis of the correlation between LDLR expression and HCC, which revealed a significant upregulation of LDLR expression in HCC tissues compared to adjacent normal liver tissues, and was negatively correlated with survival (Figure 4A-B). Thereafter, we established that the mean fluorescence intensity (MFI) of CD8^+^ T cell LDLR was higher in response to treatment with PF-06446846 (Figure 4C). To determine whether the upregulation of LDLR in response to the inhibition of PCSK9 can regulate the effector function of CD8^+^T cells, CD8^+^ T cells were isolated from the spleens of LDLR-knockout mice.

Phenotypic staining of spleen tissues revealed no significant differences in the percentage LDLR content of effector memory CD8^+^T cells between the control and PF-06446846 groups stimulated with CD3 and CD28 antibodies (Figure 4D). Phenotypic staining also revealed that LDLR was significantly upregulated upon stimulation with CD3 and CD28 antibodies (Figure 4E), whereas intracellular cytokine staining indicated that the production of granzyme B, perforin, and IFN-γ was significantly reduced in response to the knockout of LDLR in CD8 T cells (Figure 4F-H). Furthermore, CTLs in which LDLR had been overexpressed were found to be characterized by significant increases in the production of granzyme B and TNF-α^22^. These results confirm that the inhibition of PCSK9 contributes to modifying the anti-HCC function of CD8 T cells by regulating the expression of LDLR.

### LDLR is involved in CD8^+^ T cell activation by regulating the mTORC1 pathway

**Figure 5:**
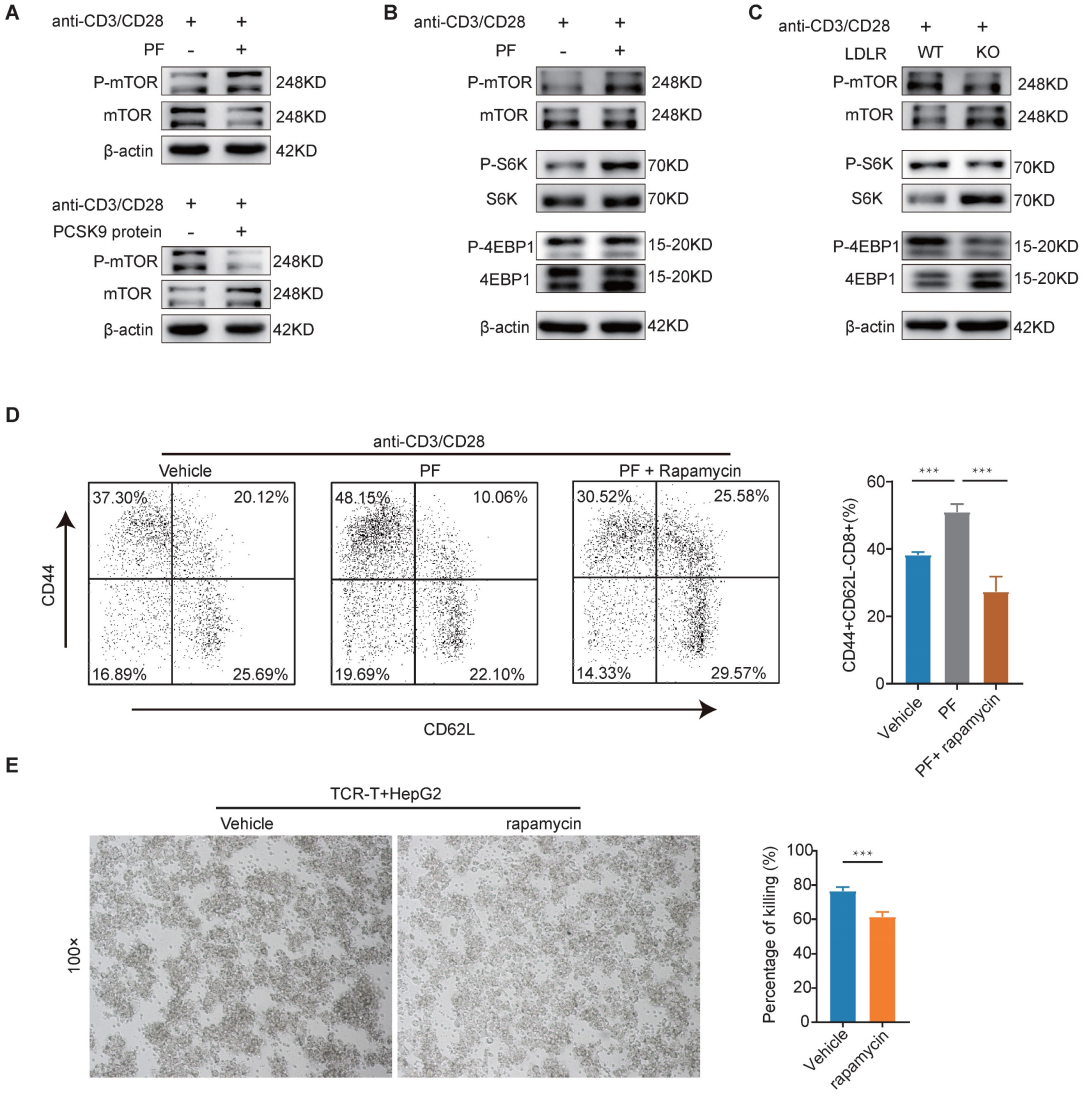
** Low-density lipoprotein receptors are involved in CD8^+^ T cell activation by regulating the mTORC1 pathway.** (A) Western blot analysis performed to detect the phosphorylation of mTOR in CD8 TCR-T cells treated with PF-06446846 or PCSK9 recombinant protein (5 μg/mL) under stimulation with 1 μL/mL anti-CD3 an CD28 antibodies for 12 h. (B) Western blot analysis performed to detect the phosphorylation of mTOR, S6K. and 4EBP1 in wild-type (WT) CD8 T cells treated with PF-06446846 under stimulation with 1μg/mL anti-CD3 and anti-CD28 antibodies for 12 h. (C) Western blot analysis performed to detect the phosphorylation of mTOR, S6K, and 4EBP1 in WT CD8 T cells and LDLR KO CD8 T cells under stimulation with 1 μg/mL anti-CD3 and anti-CD28 antibodies for 12 h. (D) WT CD8 T cells were treated with or without PF-06446846 and rapamycin (0.1 μM) for 24 h, and memory phenotypes (CD44 and CD62L) were determined by flow cytometry. (E) AFP TCR-T cells were pre-treated with rapamycin for 12 h (0.1 μM), then co-cultured with TCR-T cells for 24 h (E: T = 1:1). Data are presented as the means ± SEM. *P < 0.05, **P < 0.01, ***P < 0.001, ****P < 0.0001, NS, not significant.

Downregulation of PCSK9 by PF-06446846 promoted the expression of membrane LDLR in human CD8 TCR-T (Figure S4A-B) and mouse CD8 T cells (Figure 4C and Figure S4C). Upon recognition of antigens, naive CD8 T cells exhibit robust proliferation, resulting in the generation and activation of effector CTLs^23^, and it has been established that the activation of mTORC1 is essential for facilitating the response of CD8 effector T cells^24^. To characterize the involvement of mTOR in this response, we initially examined the levels of mTOR phosphorylation in human CD8^+^ TCR-T cells. We found PF-06446846 promoted an increase phosphorylation of mTOR in human CD8^+^ TCR-T cells, treatment with PCSK9 recombinant protein had the opposite effect, causing a reduction in mTOR phosphorylation (Figure 5A). Subsequently, CD8^+^ T cells were isolated from WT mouse spleens and upon stimulation with PF-06446846, we observed enhanced phosphorylation of mTORC1 pathway proteins including mTOR, S6 kinase (S6K), and 4E-binding protein 1 (4EBP-1) (Figure 5B). Here, we isolated the CD8 T cells from LDLR^-/-^ mouse spleen, and detected the membrane LDLR expression (Figure S4D). In contrast, the phosphorylation of mTOR, S6K, and 4EBP-1 was lower in activated LDLR^-/-^CD8^+^ T cells than in WT CD8^+^ T cells (Figure 5C). Further experiments revealed that an mTOR inhibitor blocked the PF-06446846-mediated upregulation of CD44^+^CD62L^-^CD8 T cells (Figure 5D), and that pre-treatment of TCR-T cells with rapamycin decreased the killing function of these cells *in vitro* (Figure 5E).

### The LDLR-mediated uptake of LDL-cholesterol is associated with mTORC1 activation in CD8^+^ T cells

**Figure 6:**
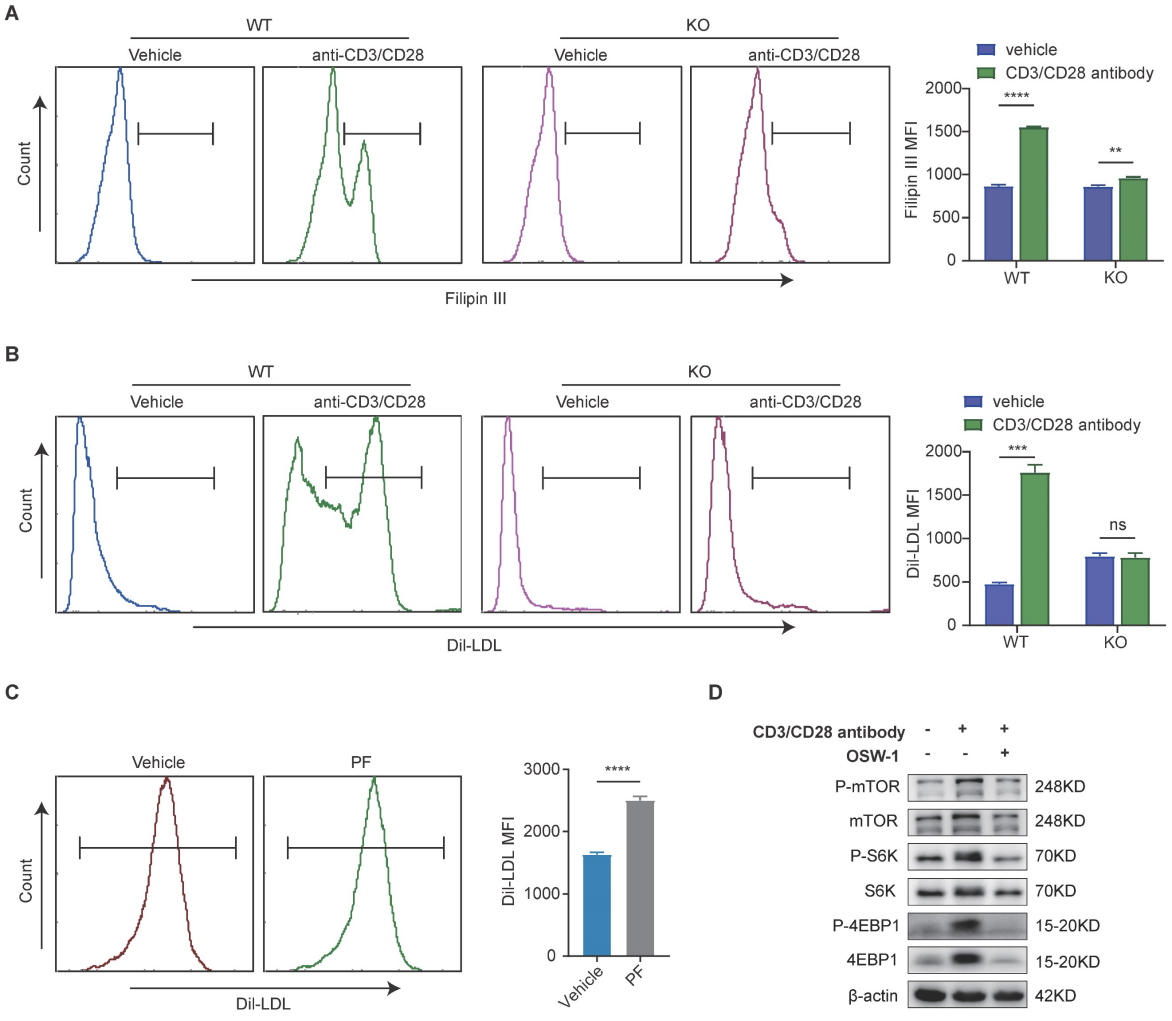
** Low-density lipoprotein receptor-mediated uptake of low-density lipoprotein-cholesterol is associated with mTORC1 activation in CD8 T cells.** (A) Filipin III staining analysis of the cholesterol content of wild-type (WT) CD8 T cells and low-density lipoprotein receptor (LDLR) KO CD8 T cells with or without anti-CD3 and anti-CD28 antibody stimulation for 24 h. (B) Uptake of DiL-LDL in WT CD8 T and LDLR KO CD8 T cells was analyzed with or without anti-CD3 and anti-CD28 antibodies for 24 h. (C) Flow cytometric analysis of DiL-LDL MFI of WT CD8T cells with or without PF-06446846 under stimulation with 1 μg/mL anti-CD3 and anti-CD28 antibodies for 24 h. (D) Western blot analysis performed to detect the effects of OSBP1 inhibition by OSW-1 (20 nM) on activation of the mTORC1 pathway in CD8 T cells under stimulation with anti-CD3 and anti-CD28 antibodies for 12 h. Data are presented as the means ± SEM. *P < 0.05, **P < 0.01, ***P < 0.001, ****P < 0.0001, NS, not significant.

Considering the pivotal role of cholesterol in CD8 T cell activation and proliferation, we went on to examine whether a defective LDLR-dependent uptake of LDL-cholesterol would impair the phosphorylation of mTOR in CD8 T cells. Following T cell activation, we observed that the levels cholesterol in WT CD8 T cells were higher than those in LDLR^-/-^CD8 T cells (Figure 6A). Thereafter, we investigated whether the impaired cholesterol metabolism in LDLR^-/-^CD8 T cells is due to reduced LDL uptake. The PE labeling of LDL revealed higher levels of uptake in WT CD8 T cells than in LDLR^-/-^CD8 T cells (Figure 6B). Furthermore, treatment with PF-06446846 was observed to promote the uptake of LDL by CD8 T cells in response to stimulation with CD3 and CD28 antibodies (Figure 6C). In this regard, the findings of a previous study have indicated that LDL-cholesterol is trafficked to the lysosomal membrane for the activation of mTOR, and that OSBP is essential for the activation of mTORC1 via LDL-cholesterol transport^25^. Consistently, we demonstrated that the OSBP inhibitor OSW-1 promoted a significant attenuation of the anti-CD3/CD28-mediated activation of the mTORC1 pathway of CD8 T cells (Figure 6D). Collectively, the findings indicate that the LDLR-mediated uptake of LDL cholesterol play a pivotal role in the metabolic support and activation of CD8 T cells, which in turn regulate the activation of the mTORC1 pathway.

### The PCSK9 inhibitor enhances the anti-HCC efficacy of adoptive T-cell therapy and PD-1 antibody

**Figure 7:**
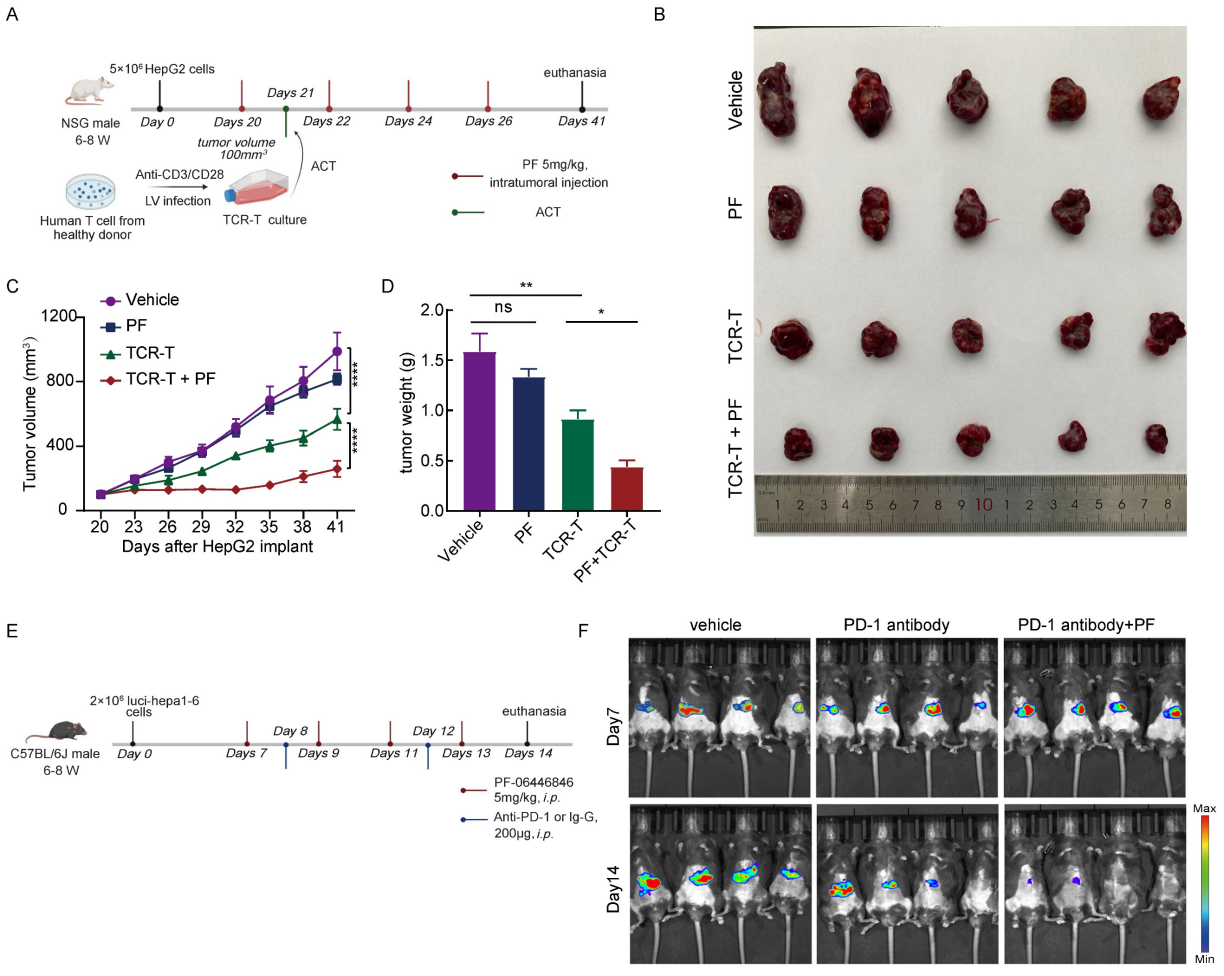
** Inhibition of PCSK9 enhances the anti-HCC efficacy of adoptive T-cell therapy and PD-1 antibody.** (A) Illustration of the adoptive transfer of 5 million TCR-T cells or MOCK-T cells to HepG2 tumor-bearing NPG mice treated with PF-06446846 (5 mg/kg) or vehicle. (B) Representative image of the HepG2 tumors that developed in the mice of each experimental group. The growth (C) and weight (D) of tumors that developed in HepG2 tumor-bearing NPG mice (n = 5). (E) Illustration of the administration of PF-06446846 (5 mg/kg) and PD-1 antibody to luci-hepa1-6 tumor-bearing C57BL/6J mice. (F) Luciferase imaging. Data are presented as the means ± SEM. *P < 0.05, **P < 0.01, ***P < 0.001, ****P < 0.0001, NS, not significant.

To further evaluate the anti-HCC effects the PCSK9 inhibitor PF-06446846, we treated NSG mice bearing HepG2 tumors with PF-06446846 and inoculated these mice with 5×10^6^ AFP-specific TCR-T cells (Figure 7A). We found that combination therapy with PF-06446846 and TCR-T cells resulted in a smaller tumor volume and weight than in mice inoculated with TCR-T cells alone (Figure 7B-D). We then investigated the PD-1 expression of transferred T cells by sacrificing mice at day 7 after TCR-T transferring (Figure S5A). We found that PF-06446846 down-regulated PD-1 expression of tumor-infiltrating CD8^+^ TCR-T (Figure S5B-C). To gain further insights from the perspective of potential therapeutic strategies, we investigated the impact of PF-06446846 on anti-PD-1 antibody activity. Specifically, luci-hepa1-6 HCC cells were generated and subsequently injected into the left lobes of C57BL/6 mice's liver (Figure 7E). In line with expectations, we found that targeting PCSK9 enhanced the tumor response to anti-PD-1 immunotherapy (Figure 7F). The results thus suggest that the inhibition of PCSK9 augments the therapeutic efficacy of adoptive T-cell therapy and PD-1 antibody against HCC.

## Discussion

By yielding essential membrane components and metabolites, the metabolism of cholesterol make important contributions to a diverse range of biological functions, and by manipulating cholesterol metabolism it is feasible to inhibit tumor growth and reinvigorate anti-tumor immunity^26^. However, the cholesterol content of infiltrating immune cells is unevenly distributed, and the HCC microenvironment is characterized by a pronounced inhibition of cholesterol metabolism in CD8^+^ T cells and induces dysfunction and exhaustion^27^. Consequently, tumor-infiltrating CD8 T cells need to acquire cholesterol to maintain their tumor-killing functions. Previous studies in this regard have demonstrated that the inhibition of cholesterol esterification not only effectively inhibits HCC development^28^ but also enhances the T cell-mediated anti-tumor immune response^22^, ^29^, and relevant studies have also documented that the inhibition of PCSK9 can promote anti-tumor effects by reprogramming cholesterol metabolism in a range of cancer types^12^. However, it remains unclear as to whether PCSK9 influences the T cell-mediated immune responses in HCC.

In this study, we established that high levels of PCSK9 expression are associated with a reduced OS and T-cell infiltration in HCC. Subsequently, we demonstrated that inhibition of PCSK9 promoted anti-HCC effects by enhancing the response of CD8^+^ T cells. It has previously been established that the main biological function of PCSK9 is to bind LDLR and thereby target it for lysosomal degradation^30^, and accordingly, in this study, we investigated the correlation between LDLR expression and overall survival in patients with HCC. We found that the expression of LDLR was significantly lower in HCC samples, and that compared with a high expression, a low expression of LDLR was associated with shorter OS time. In this regard, a recent study has reported that low LDLR expression is associated with the promotion of HCC cell proliferation and metastasis^31^. Mechanistically, the inhibition of PCSK9 promotes an upregulation of the LDLR of CD8 T cells. In response to the knockout of LDLR in CD8 T cells, we detected no significant difference between control and PF-06446846 groups with respect the percentage CD44^+^CD62L^-^ effector memory in response to stimulation. The activation of CD8 T cells requires the upregulation of LDLR, and in LDLR^-/-^CD8 T cells, we detected significant reductions in the production of granzyme B, perforin, and IFN-γ. Treatment with PF-06446846 was demonstrated to activate the mTORC1 pathway by upregulating LDLR, whereas increases in the percentage of CD44^+^CD62L^-^CD8 T cells promoted by PF-06446846 were blocked by treatment with rapamycin. In this context, recent evidence indicates that the mTORC1 pathway plays an essential role in the activation of CD8 T cells^24^, ^32^, ^33^, and the activation of this pathway is demonstrated to augment the antitumor functionality of CD8 T cells^34^, ^35^. On the basis of these findings, it appears that activated CD8 T cells acquire the necessary amounts of cholesterol via exogenous uptake rather than by endogenous synthesis. Thus, the LDLR-mediated uptake of cholesterol provides metabolic support for the activation CD8 T cells, and hence contributes to promoting their anti-tumor functions. LDLR-mediated cholesterol uptake directly activates lysosomal mTORC1 in CD8 T cells.

Collectively, our findings in this study indicate that the inhibition of PCSK9 enhances the anti-HCC function of CD8 T cells. Notably, we found that treatment with PF-06446846, an inhibitor of PCSK9, enhanced the anti-HCC function of adoptive T-cell therapy and PD-1 antibodies. These findings will accordingly provide a basis for developing a combined metabolic intervention strategy that could be adopted to enhance the efficacy of immunotherapy for patients with HCC.

## Supplementary Material

Supplementary figures.
