# Supplementary material for: Inhibition of PCSK9 enhances the anti-hepatocellular carcinoma effects of TCR-T cells and anti-PD-1 immunotherapy
Source: Int J Biol Sci. 2024 Jul 15;20(10):3942–55. doi: 10.7150/ijbs.93668 (PMC11302870; doi:10.7150/ijbs.93668)
Supplement: Supplementary file 1 — Supplementary figures. [file ijbsv20p3942s1.pdf]

Figure S1. GEO database analysis of the correlation between PCSK9 mRNA expression and immunocytes in HCC. (A) CD4. (B) NK cell makers: CD16, CD56. (C) macrophage cell makers: CD68, CD80 and CD206.

Figure S2. The Effects of PF-06446846 on the Cell Viability of HCC cell lines and Establishment of AFP TCR-T cells. (A) Cell viability of HepG2, Huh7, PLC/PRF/5 and snu449 cells after PF-06446846 for 24 h. (B) Cell viability of TCR-T cells after PF-06446846 for 24 h. (C) Analysis of PCSK9 and AFP in HepG2 by Western blot after PF-06446846 for 24 h. (D) Schematic representation of the lentiviral vectors for AFP TCR-T cells. (E) Flow cytometry analysis of the percentage of AFP-specific TCR T cells. (F) (G) Verification of the AFP TCR-T killing function. Data are presented as the means ± SEM. *$P < 0.05$, **$P < 0.01$, ***$P < 0.001$, ****$P < 0.0001$, NS, not significant.

Figure S3. Anti-HCC effects induced by the PF-06446846 are dependent on CD8 T cells in vivo. (A-B) The photos of the tumor masses, body weight and tumor weight. (C) Percentage of $CD3^+CD8^+$ T cells in mouse blood determined by flow cytometric analysis. *$P < 0.05$, **$P < 0.01$, ***$P < 0.001$, ****$P < 0.0001$, NS, not significant.

Figure S4. Inhibition of PCSK9 up-regulated LDLR in CD8 T cells. (A) Analysis of PCSK9 in CD8 TCR-T cells by Western blot after PF-06446846 for 12 h. (B) Flow cytometry analysis of membrane LDLR expression in CD8 TCR-T cells after PF-06446846 for 12 h. (C) Analysis of PCSK9 in mouse CD8 T cells by Western blot after PF-06446846 for 12 h. (D) Flow cytometry analysis of membrane LDLR expression in mouse CD8 T cells after PF-06446846 for 12 h. *$P < 0.05$, **$P < 0.01$, ***$P < 0.001$, ****$P < 0.0001$, NS, not significant.

Figure S5. The PD-1 expression of tumor infiltrating TCR-T after PF-06446846 treatment in vivo. (A) Illustration of the adoptive transfer of 5 million TCR-T cells or MOCK-T cells to HepG2 tumor-bearing NPG mice treated with PF-06446846 (5 mg/kg) or vehicle, mice were sacrificed 7 days after transferring and the cells in the tumor were analyzed. (n = 4 mice per group). (B-C) The percentage of $PD\text{-}1^+$CD8 TCR-T cells in tumor-infiltrating total CD8 TCR-T of each mouse 7 days after TCR-T transfer was shown (n = 4). *$P < 0.05$, **$P < 0.01$, ***$P < 0.001$, ****$P < 0.0001$, NS, not significant.

Figure S1

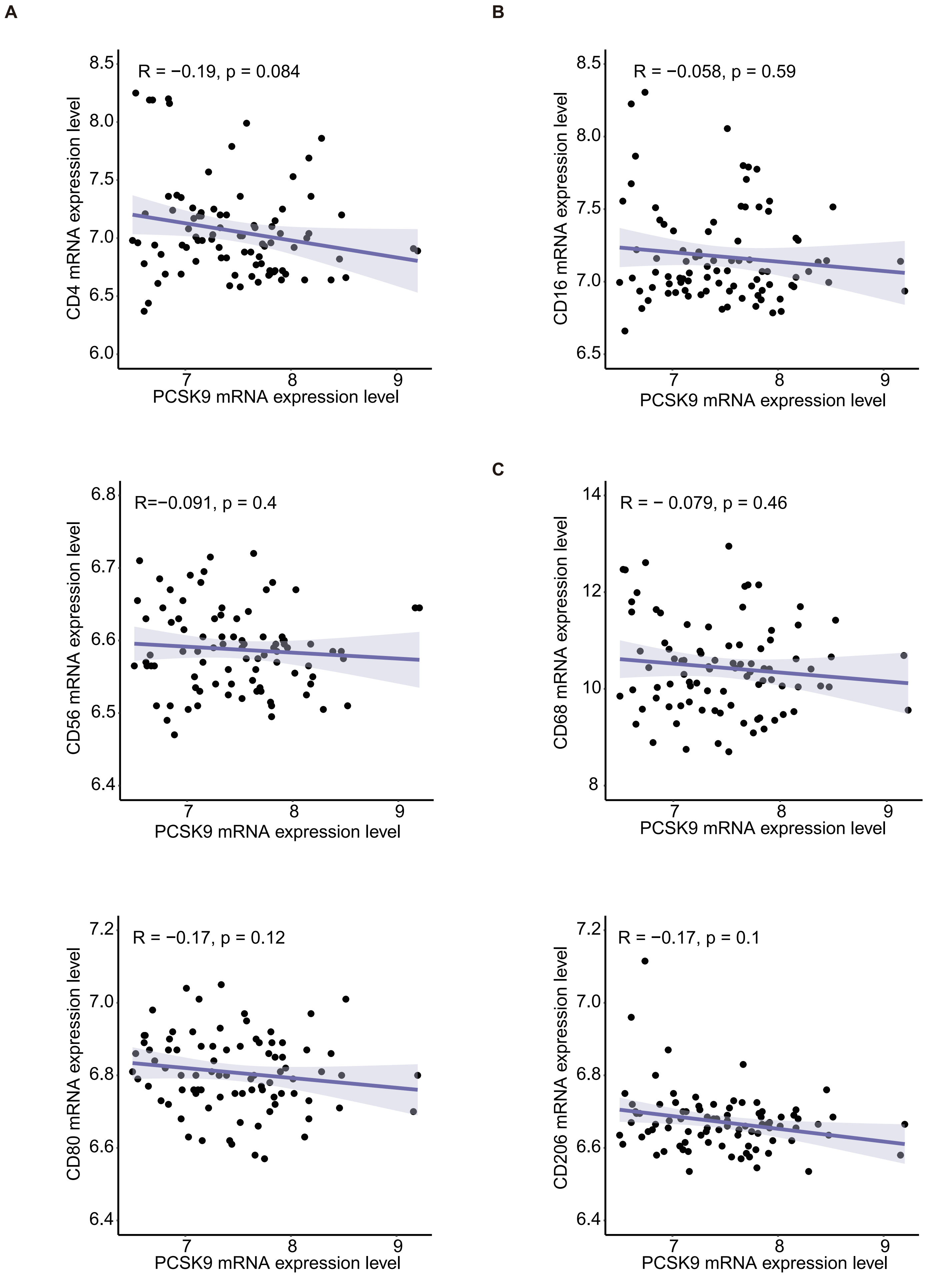
A
B
C
R = −0.19, p = 0.084
CD4 mRNA expression level
PCSK9 mRNA expression level
R = −0.058, p = 0.59
CD16 mRNA expression level
PCSK9 mRNA expression level
R=−0.091, p = 0.4
CD56 mRNA expression level
PCSK9 mRNA expression level
R = − 0.079, p = 0.46
CD68 mRNA expression level
PCSK9 mRNA expression level
R = −0.17, p = 0.12
CD80 mRNA expression level
PCSK9 mRNA expression level
R = −0.17, p = 0.1
CD206 mRNA expression level
PCSK9 mRNA expression level

Figure S2

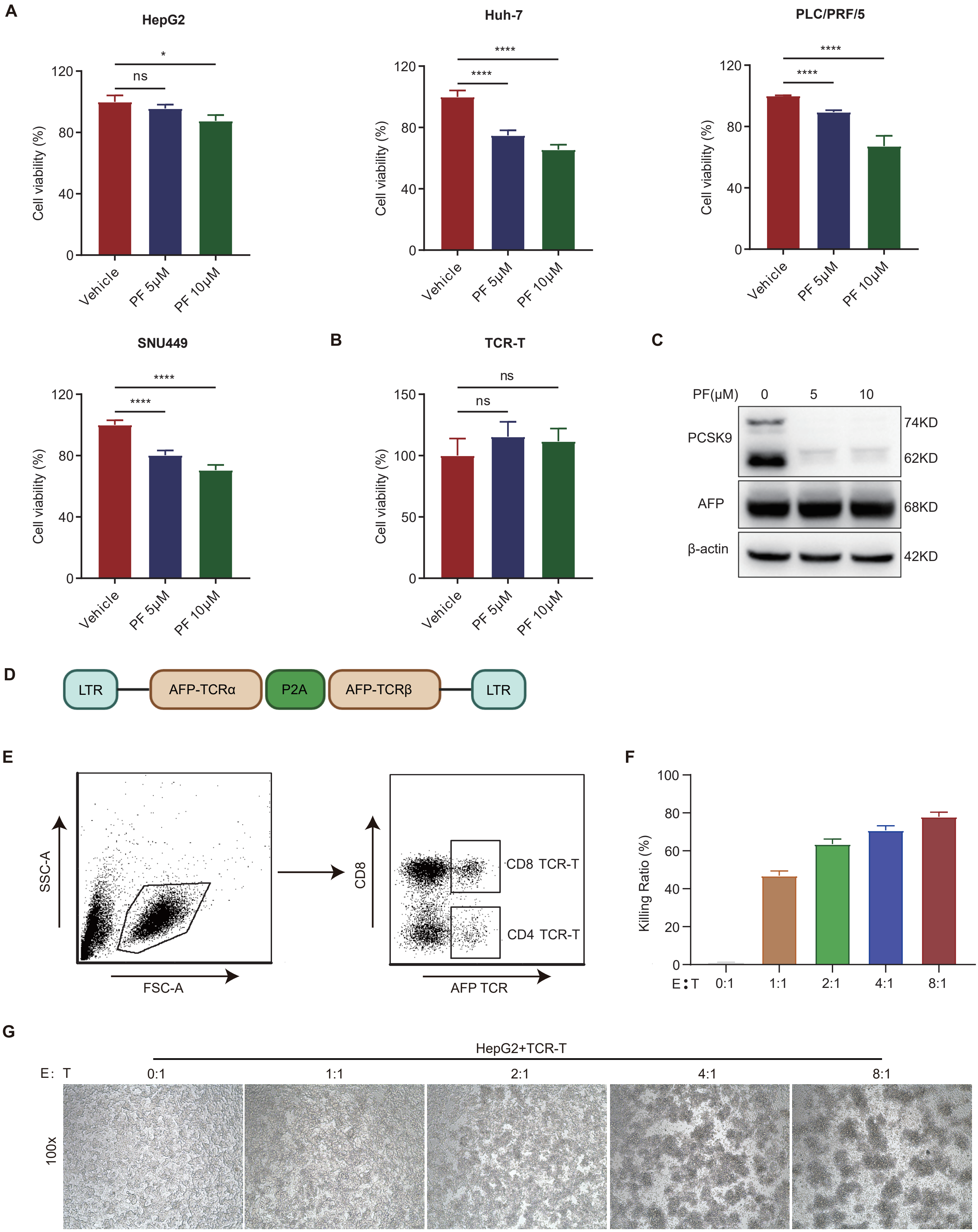
A
HepG2
Huh-7
PLC/PRF/5
SNU449
Cell viability (%)
Vehicle
PF 5μM
PF 10μM
ns
*
****
B
TCR-T
C
PF(μM) 0 5 10
PCSK9
AFP
β-actin
74KD
62KD
68KD
42KD
D
LTR
AFP-TCRα
P2A
AFP-TCRβ
LTR
E
SSC-A
FSC-A
CD8
AFP TCR
CD8 TCR-T
CD4 TCR-T
F
Killing Ratio (%)
E：T 0:1 1:1 2:1 4:1 8:1
G
HepG2+TCR-T
E: T 0:1 1:1 2:1 4:1 8:1
100x

Figure S3

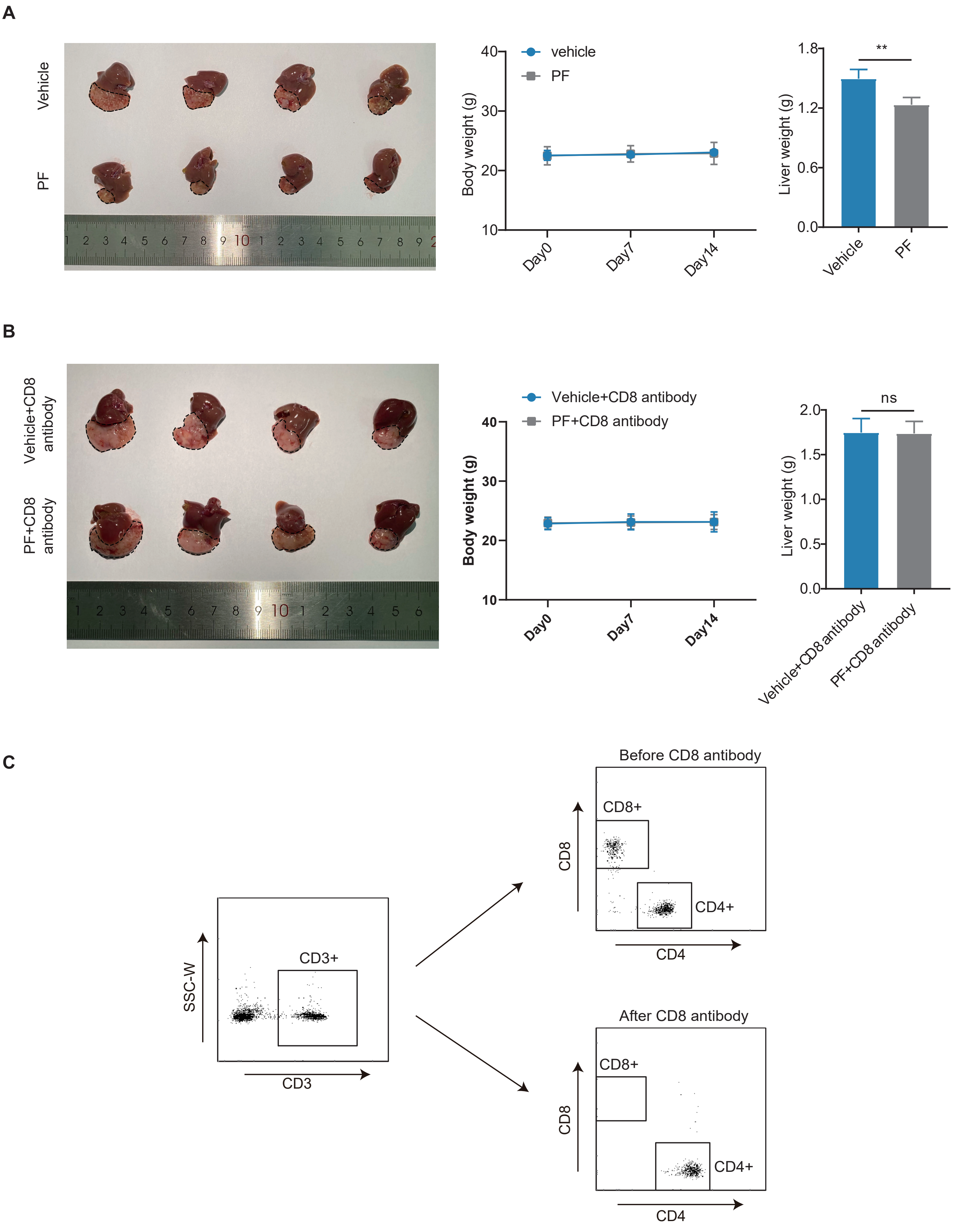
A
Vehicle
PF
vehicle
PF
Body weight (g)
Day0
Day7
Day14
Liver weight (g)
**
Vehicle
PF
B
Vehicle+CD8 antibody
PF+CD8 antibody
Body weight (g)
Day0
Day7
Day14
Liver weight (g)
ns
Vehicle+CD8 antibody
PF+CD8 antibody
C
SSC-W
CD3+
CD3
Before CD8 antibody
CD8+
CD4+
CD8
CD4
After CD8 antibody
CD8+
CD4+
CD8
CD4

Figure S4

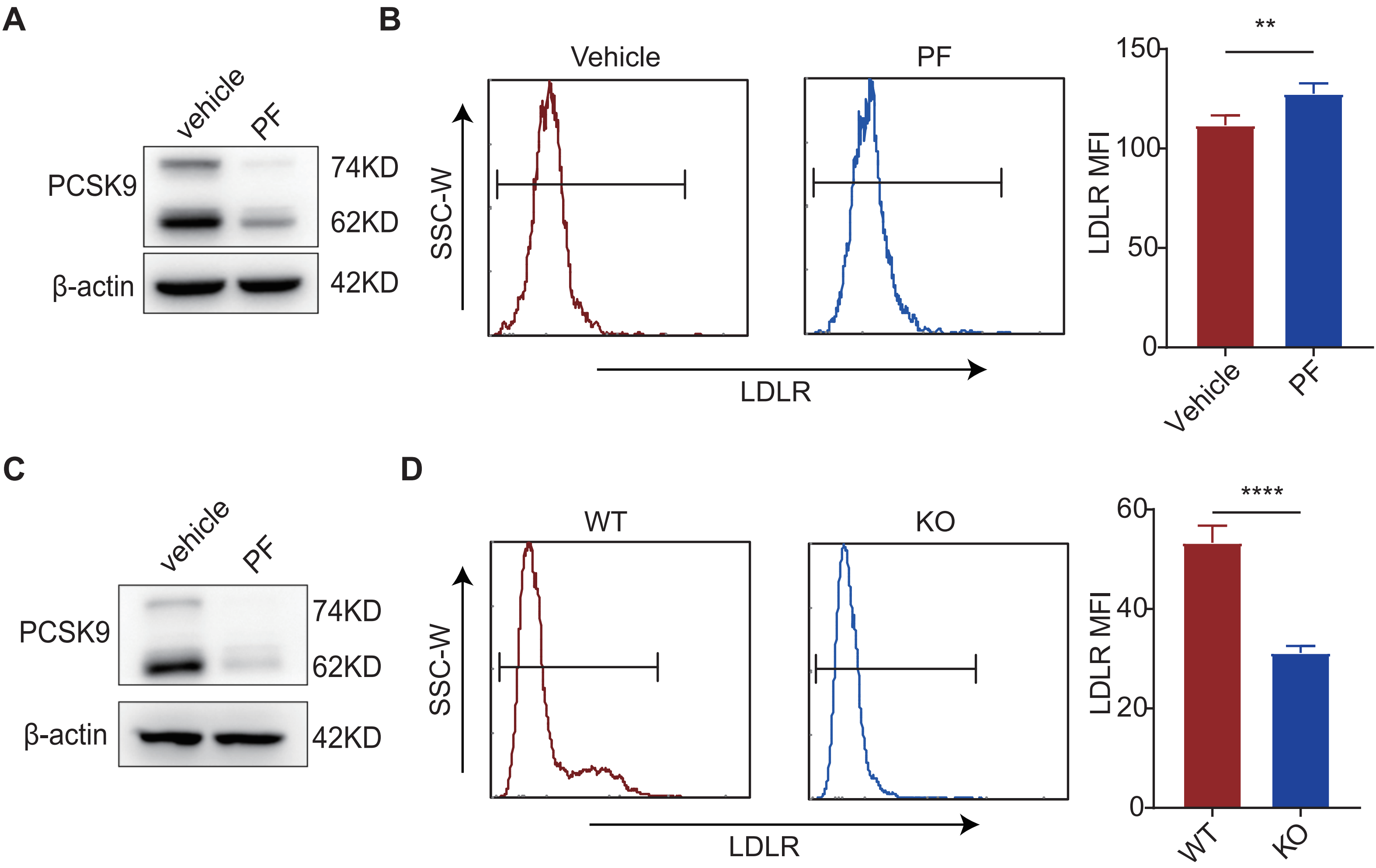

A
vehicle
PF
PCSK9
74KD
62KD
β-actin
42KD
B
Vehicle
PF
SSC-W
LDLR
**
150
100
50
0
LDLR MFI
Vehicle
PF
C
vehicle
PF
PCSK9
74KD
62KD
β-actin
42KD
D
WT
KO
SSC-W
LDLR
****
60
40
20
0
LDLR MFI
WT
KO

Figure S5

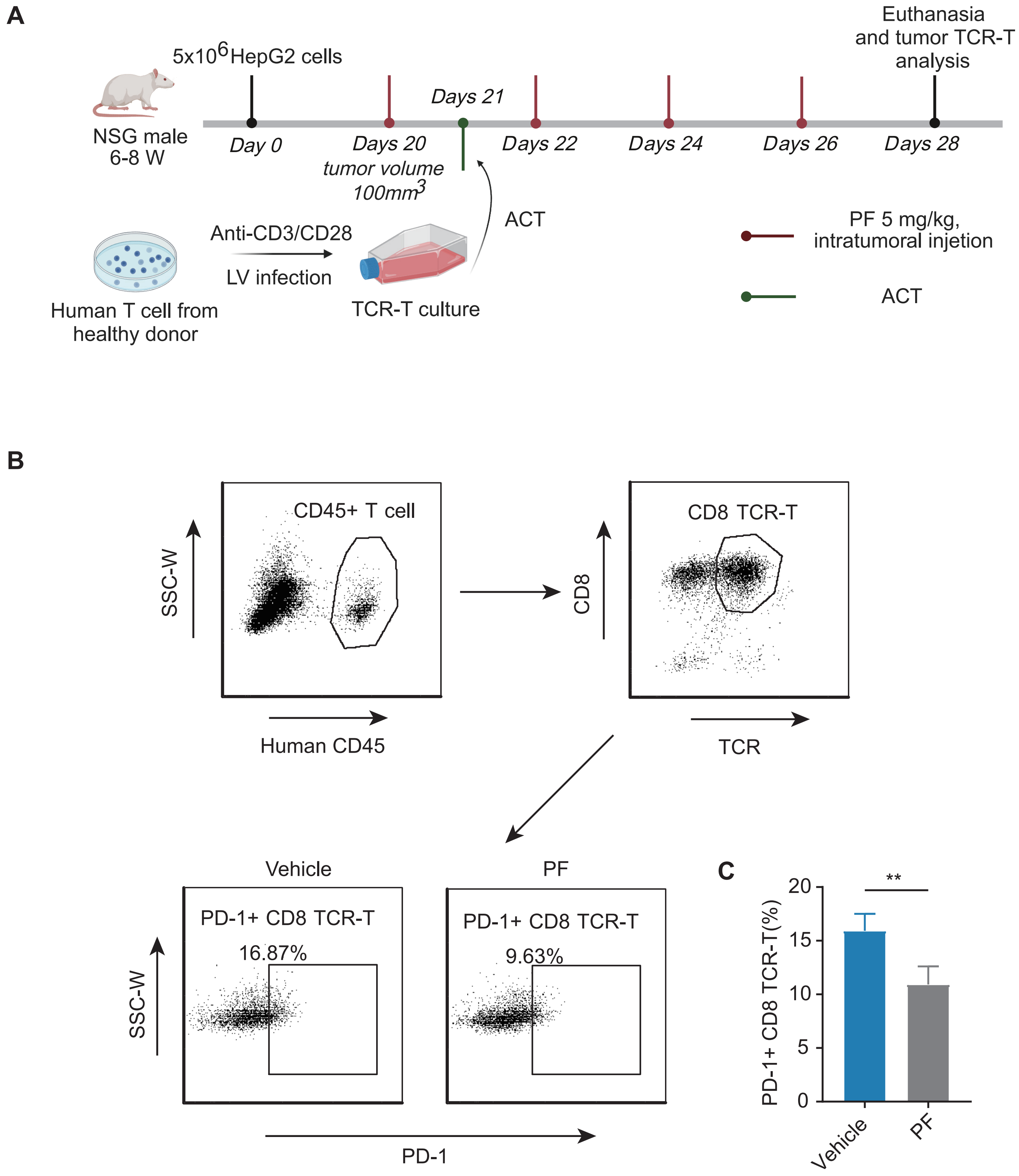
A
5x10^6 HepG2 cells
Euthanasia and tumor TCR-T analysis
NSG male 6-8 W
Day 0
Days 20 tumor volume 100mm3
Days 21
Days 22
Days 24
Days 26
Days 28
ACT
Anti-CD3/CD28
LV infection
Human T cell from healthy donor
TCR-T culture
PF 5 mg/kg, intratumoral injetion
ACT
B
CD45+ T cell
SSC-W
Human CD45
CD8 TCR-T
CD8
TCR
Vehicle
PF
PD-1+ CD8 TCR-T
16.87%
PD-1+ CD8 TCR-T
9.63%
SSC-W
PD-1
C
PD-1+ CD8 TCR-T(%)
20
15
10
5
0
**
Vehicle
PF
